# Negative Differential Conductance & Hot-Carrier Avalanching in Monolayer WS2 FETs

**DOI:** 10.1038/s41598-017-11647-6

**Published:** 2017-09-12

**Authors:** G. He, J. Nathawat, C.-P. Kwan, H. Ramamoorthy, R. Somphonsane, M. Zhao, K. Ghosh, U. Singisetti, N. Perea-López, C. Zhou, A. L. Elías, M. Terrones, Y. Gong, X. Zhang, R. Vajtai, P. M. Ajayan, D. K. Ferry, J. P. Bird

**Affiliations:** 1 1Department of Electrical Engineering, University at Buffalo, the State University of New York, Buffalo, NY 14260-1900 USA; 2 Department of Physics, University at Buffalo, the State University of New York, Buffalo, NY 14260-1500 USA; 3 Department of Physics, King Mongkut’ s Institute of Technology Ladkrabang, Bangkok, 10520 Thailand; 4 High-Frequency High-Voltage Device and Integrated Circuits Center, Institute of Microelectronics of Chinese Academy of Sciences, 3 Beitucheng West Road, Chaoyang District, Beijing, PR China; 5 Department of Physics and Center for 2-Dimensional and Layered Materials, The Pennsylvania State University, University Park, Pennsylvania, 16802 USA; 6 Department of Materials Science and Engineering and Materials Research Institute, The Pennsylvania State University, University Park, Pennsylvania, 16802 USA; 7 Department of Chemistry and Materials Research Institute, The Pennsylvania State University, University Park, Pennsylvania, 16802 USA; 8 Department of Materials Science and Nano Engineering, Rice University, Houston, TX 77005 USA; 9 School of Electrical, Computer, and Energy Engineering, Arizona State University, Tempe, Arizona 85287-5706 USA

## Abstract

The high field phenomena of inter-valley transfer and avalanching breakdown have long been exploited in devices based on conventional semiconductors. In this Article, we demonstrate the manifestation of these effects in atomically-thin WS_2_ field-effect transistors. The negative differential conductance exhibits all of the features familiar from discussions of this phenomenon in bulk semiconductors, including hysteresis in the transistor characteristics and increased noise that is indicative of travelling high-field domains. It is also found to be sensitive to thermal annealing, a result that we attribute to the influence of strain on the energy separation of the different valleys involved in hot-electron transfer. This idea is supported by the results of ensemble Monte Carlo simulations, which highlight the sensitivity of the negative differential conductance to the equilibrium populations of the different valleys. At high drain currents (>10 μA/μm) avalanching breakdown is also observed, and is attributed to trap-assisted inverse Auger scattering. This mechanism is not normally relevant in conventional semiconductors, but is possible in WS_2_ due to the narrow width of its energy bands. The various results presented here suggest that WS_2_ exhibits strong potential for use in hot-electron devices, including compact high-frequency sources and photonic detectors.

## Introduction

The emergence, in recent years, of monolayer transition-metal dichalcogenides has opened up broad possibilities for the realization of functional electronic and optoelectronic devices^1, 2^ In contrast to graphene, many of these materials (including MoS_2_, WS_2_, and WSe_2_) are semiconducting and exhibit a significant forbidden gap (>1 eV), making them well suited for use in various transistor schemes. This has driven a concerted effort to characterize the operational characteristics of such devices, with particular emphasis being placed on evaluating the efficiency (ON/OFF ratio) and sharpness (sub-threshold slope) of transistor switching, and on identifying the different scattering mechanisms that limit the channel mobility^1^ Much less attention appears to have been paid, however, to a potentially exciting application of these materials, namely the idea of using them to realize active mm-wave/terahertz sources, which derive their functionality from the dynamic transfer of hot electrons between different regions of their band structure.

Transferred-electron effects have long been exploited in conventional semiconductors, with the most well-known example being provided by the Gunn effect^3, 4^ In this phenomenon, the scattering of hot electrons from a central, high-mobility, valley, to a nearby one with heavier effective mass, gives rise to negative differential conductance and to the formation of traveling high-field domains. The domains are injected from the cathode of the device and drift towards the anode, at which point they collapse, allowing a new domain to again be injected from the cathode. The periodic repetition of this process, on a time scale determined by the saturated drift velocity and by the anode-cathode separation, results in the emission of (microwave) radiation from the device, a phenomenon that has been exploited for some five decades in compact, inexpensive microwave sources^4^

**Figure 1:**
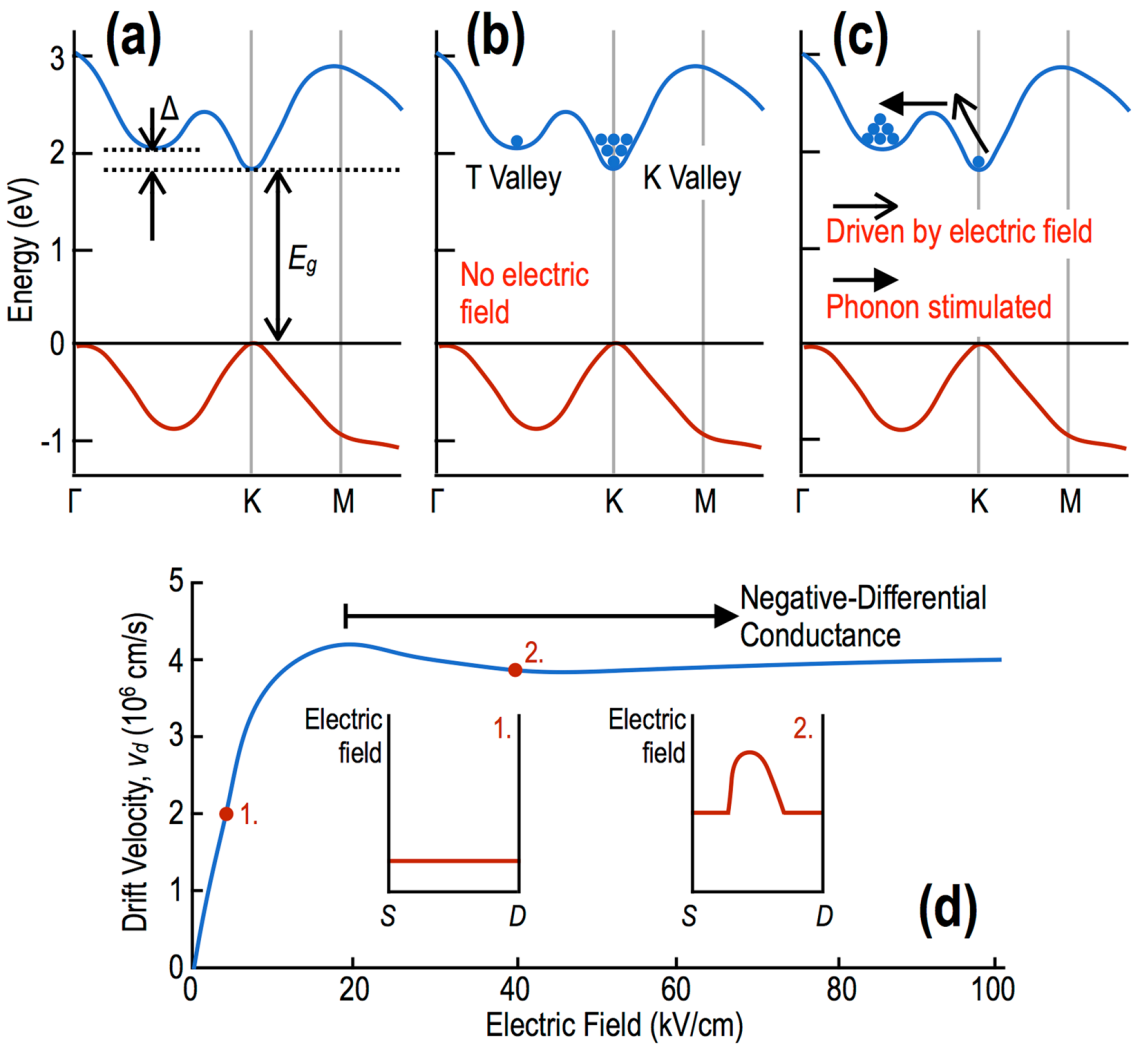
Bandstructure of WS2 and the mechanism for its negative differential conductance. (**a**) Band structure of monolayer WS_2_. The T valley is located between the Γ and K points, and is separated from the K valley by an amount Δ. (**b**) Relative populations of the K and T valleys is indicated schematically for monolayer WS_2_ at thermal equilibrium. (**c**) The populations of the K and T valleys change significantly under the application of an electric field. (**d**) Anticipated form of the velocity-field characteristic of monolayer WS_2_, indicating a region of negative differential velocity/conductance. The insets are schematics that represent the electric-field distribution within the sample at very-different electric fields. 1. At low fields, the electric field is approximately constant throughout the conductor. 2. In the region where negative differential conductance occurs, a high-field domain is formed and travels repeatedly through the conductor.

The purpose of this Article is to demonstrate the possibility of exploiting the specific features of the band structure of transition-metal dichalcogenides, to realize novel transferred-electron effects. The material that we chose to focus on for this purpose is WS_2_, which, when isolated in monolayer form, exhibits a band structure that suggests the strong potential for hot-electron transfer^5^ This form of this structure is shown in Fig. 1(a), which reveals the presence of a direct gap (*E*_*g*_ ${\sim }$ 1.8 eV) at the K point (and, thus, at its equivalent K′ point^5^), and of a satellite valley that is located at the “T” point between the Γ and K points. The energy separation (Δ) of these two valleys is around 80 meV (dependent upon mechanical strain, see the discussion below), and the electron mass in the satellite valley (*m*_T_^*^ = 0.75*m*_*o*_, where *m*_*o*_ is the free-electron mass) is heavier than that near the K point (*m*_K_^*^ = 0.32*m*_*o*_). (In reciprocal space, the primitive Brillouin zone cell encloses six such T valleys, in addition to a single K and a single K′ point.) This bandstructure is therefore reminiscent of that found in GaAs, the prototypical “Gunn material”, thus suggesting the possibility of achieving novel transferred-electron effects in WS_2_. More specifically, in thermal equilibrium at room temperature, we expect most of the conduction-band carriers to populate the K (K′) valleys, with a much smaller number of electrons being present in the T valleys (Fig. 1(b)). By applying a sufficiently large electric field, however, it should be possible to drive carriers up the K valleys, allowing them to eventually scatter into the lower-mobility T valleys (Fig. 1(c)). This should give rise to a region of negative slope (Fig. 1(d)) in the velocity-fiel_*d*_ (*v*_*d*_-***ε***) characteristic, and lead in turn to negative differential conductance and to high-field domain formation (see the insets to Fig. 1(d)). In this Article, we present evidence of such behavior in monolayer WS_2_, which we find exhibits clear signatures of negative differential conductance and of domain-induced current instabilities. Our experimental observations are confirmed by simulations of high-field transport, implemented via a Monte Carlo approach that includes all relevant scattering processes, both within the WS_2_ and the (SiO_2_) substrate. The calculations reveal how the relative populations of the two types of valley (K & T) evolve with electric field, and capture the characteristic features of the negative differential conductance observed in our experiments. We moreover demonstrate a novel form of high-field avalanching in these devices, which we attribute to a trap-assisted inverse-Auger process. Overall, our results represent a promising first step towards the realization of compact photonic sources based on two-dimensional (2D) transition-metal dichalcogenides. The high saturation velocity associated with this material (and with other transition-metal dichalcogenides^6, 7^) bodes well for the prospect of extending the operation of these sources to frequencies approaching the terahertz range (i.e. $\ge$10  GHz).

## Results

**Figure 2:**
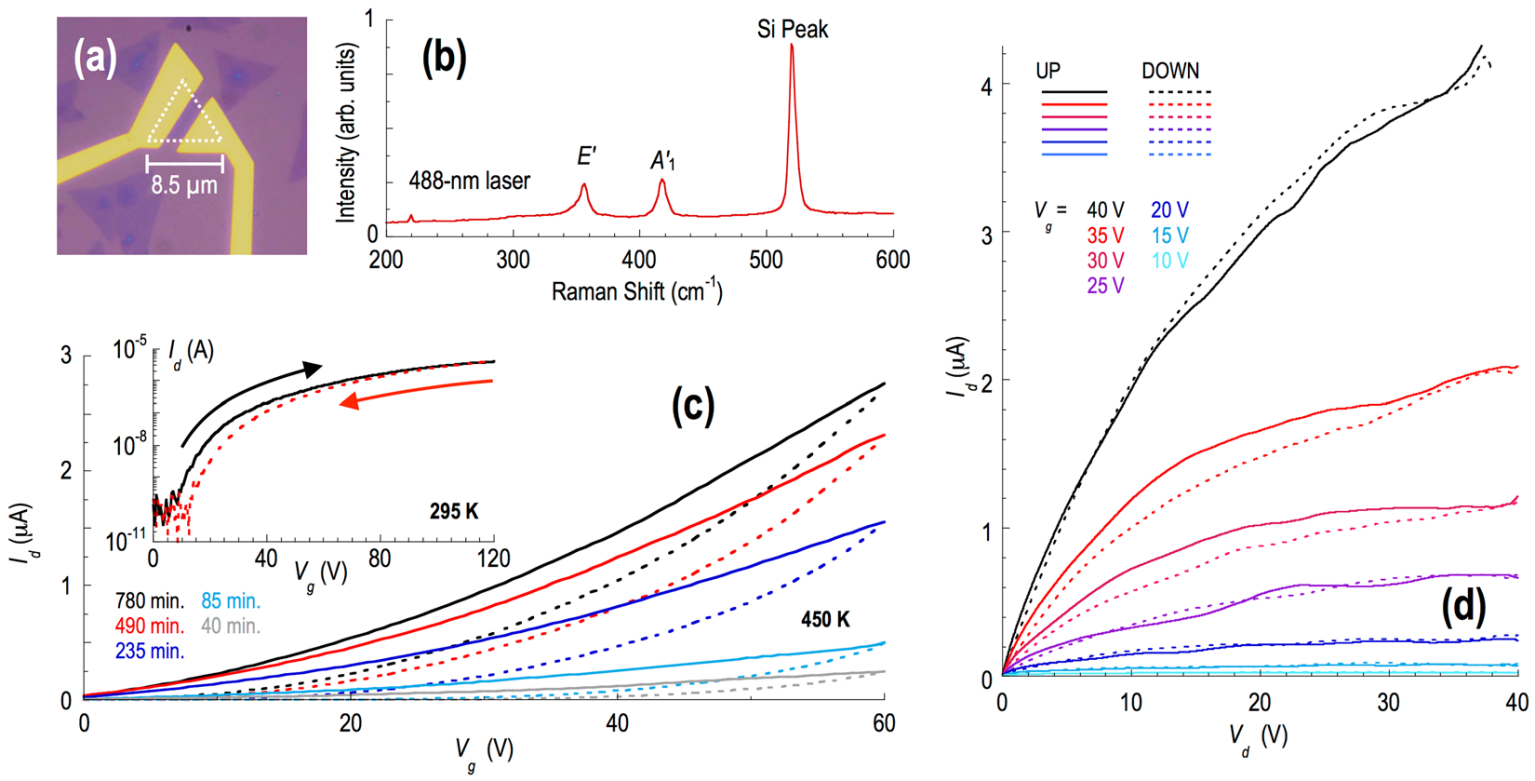
Raman spectroscopy and essential electrical characterization of the WS2 FETs. (**a**) Example of a monolayer WS_2_ transistor (Device A). The outline of the WS_2_ crystal is indicated by the white dotted line. (**b**) Raman spectrum of Device A, measured with 488-nm wavelength excitation. (**c**) The main panel plots transfer curves, measured for Device A at 450 K, after holding for that temperature for various times (indicated). The solid lines correspond to up sweeps of the gate voltage, while the dotted lines represent the corresponding down sweeps. The inset shows the transfer curve measured near room temperature, after completing the annealing process indicated in the main panel. Solid and dashed lines correspond to different sweep directions (indicated). (**d**) Transistor curves measured for Device A, after completing an annealing process similar to that indicated in Fig. 2(c). Solid and dashed lines correspond to different sweep directions (indicated).

WS_2_ crystals (Fig. 2(a)) were grown on commercial Si/SiO_2_ wafers by chemical vapor deposition (CVD) (for further details see refs 8 and 9). The monolayer character of these triangular-shaped single crystals was confirmed by Raman spectroscopy. An example is shown in Fig. 2(b), in which we plot the Raman spectrum of the device of Fig. 2(a) and find behavior consistent with previous reports for monolayer WS_2_^10–12^ The crystals were incorporated as the active channel of field-effect transistor (FET) structures, in which the variation of drain current (*I*_*d*_) could be measured as a function of the drain (*V*_*d*_) and gate (*V*_*g*_) voltages. The gating was achieved by biasing a heavily-doped Si substrate, separated from the WS_2_ crystals by a 300-nm thick SiO_2_ dielectric layer. The fabricated devices were installed in a sample chamber with a base vacuum ≥10^−6^ mbar, and which allowed for control of temperature over the range of 300–500 K. Some twenty different transistors were fabricated for the purpose of this study, with channel lengths in the range of 1–2 µm (please refer to the Supplementary Information for more details on the most heavily-studied devices, including the nomenclature that we use here to identify them). The results included in this paper have been selected for representative purposes, being typical of the behavior exhibited by the different devices.

### Negative Differential Conductance in Partially-Annealed WS2 Transistors

Recent studies of the electrical characteristics of WS_2_ FETs have focused on understanding the essential aspects of transistor operation^13–23^, and on enhancing this functionality through targeted strategies for doping^24–28^. The drive currents supported by these transistors have also been found to be significantly enhanced by thermal annealing^19^, which improves contact transparency and should also relax residual strain in the channel. In Fig. 2(c), we confirm the importance of thermal annealing by plotting various transfer curves, measured for Device A at an annealing temperature of 450 K. The annealing was performed while holding the sample chamber under vacuum, and the results of Fig. 2(c) were obtained by holding at that temperature for various times (indicated). Consistent with ref. 19, we observe a large increase in drive current when annealing over a time scale of many hours. Prior to the annealing, the current level remained in the range of 10^−9^–10^−8^ A, over the entire range of gate voltage studied. With sustained annealing, however, the current could be increased to the µA level, representing a significant improvement in performance. During annealing, the transfer curves exhibit significant hysteresis, indicating that the channel current is degraded by the influence of charge trapping. As described previously in the literature (see, for example, the discussions in ref. 29), the traps responsible for this behavior are predominantly believed to be interfacial states, present at the boundary between the channel and the gate oxide, and deep traps within gate dielectric itself. In addition to providing a pathway for the loss of carriers from the channel, the traps may also function as localized scattering sites, suppressing the drive current of the FET^1, 2^ In our experiments, the hysteresis in the transfer curves is significantly reduced at room temperature (see the inset to Fig. 2(c)), where the WS_2_ transistors exhibit an ON/OFF ratio close to 10^5^. In Fig. 2(d), we show transistor curves measured at room temperature, for another transistor (Device B) that has also been subjected to extended annealing. Consistent with the data of Fig. 2(c), we observe that the current level here rises to exceed 10 µA, at which point it approaches the saturation regime. Once again, there is a small amount of hysteresis as the drain voltage is cycled, consistent with the bias-induced excitation of carriers into/out of localized states. Importantly, however, the magnitude of this effect is small, a point that will be important to our subsequent discussions of the negative differential conductance effect.

**Figure 3:**
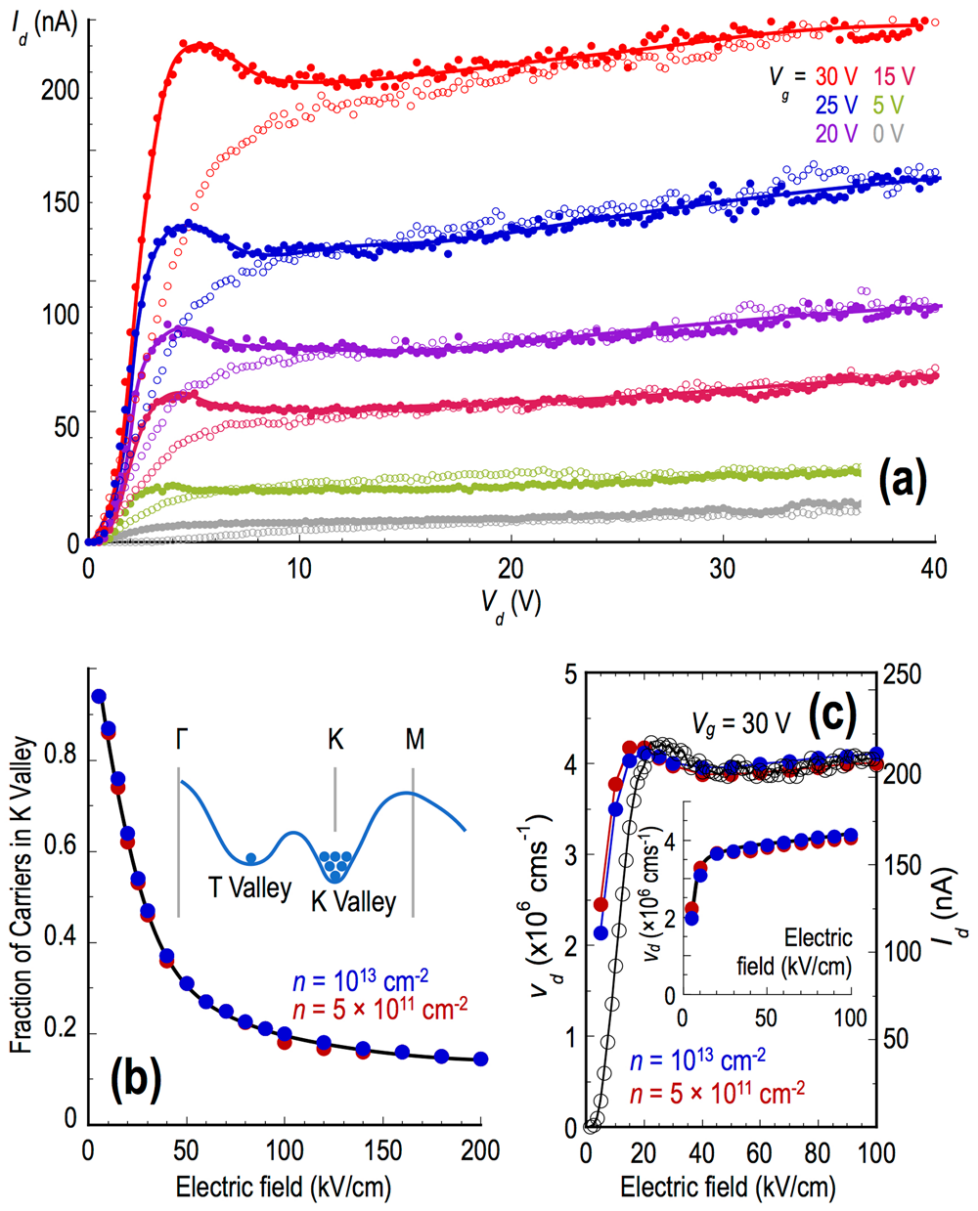
Negative differential conductance in the WS2 FETs. (**a**) Negative differential conductance in the room-temperature transistor curves (gate voltages indicated) of Device C. The filled and open symbols represent measurements taken while sweeping the drain voltage up and down from zero, respectively. The solid lines are guides to the eye that are drawn through the up sweeps to highlight the increased current noise that onsets on entering the region of negative differential conductance. (**b**) The main panel plots the carrier population in the K valley, at room temperature and at two different densities (indicated), as a function of electric field. The population decreases rapidly with increasing field, as the population of the T valley correspondingly increases. The inset indicates schematically the populations of the two valleys at thermal equilibrium. (**c**) In the main panel we compare the form of the calculated velocity-field curves, at two different densities (indicated) and for Δ = 110 meV, with the *I*_*d*_-*V*_*d*_ characteristic of Device C (the *V*_*g*_ = 30-V curve from Fig. 3(a)). Filled symbols correspond to the electron density, while the open symbols denotes the drain current. The inset shows the calculated velocity-field characteristics, for the same densities as considered in the main panel and for unstrained WS_2_ with Δ = 80 meV. Note the absence of any negative differential conductance in this case.

It is quite clear that the transistor curves of Fig. 2(d) exhibit no evidence of negative differential conductance, in spite of the fact that the electric field in the channel reaches as much as 40 kV/cm. In Fig. 3(a), however, we show a very clear example of negative differential conductance that we have observed in these devices. While these data were obtained for Device C, under conditions that are described in the following paragraph, similar examples of negative differential conductance were obtained in all devices studied (see the Supplementary Information for further examples). There are a number of aspects of the data in Fig. 3(a) that suggest that they are indeed associated with negative differential conductance arising from the influence of inter-valley transfer. The first feature that we note is the *clear region of negative differential conductance* itself, which is only observed when increasing the drain voltage, and which onsets at around *V*_*d*_ = 5 V (corresponding to a channel field of 20–30 kV/cm). Crucially, the *onset of the negative differential conductance is accompanied by a marked increase in the noise level* in the drain current, a characteristic that commonly signals the formation of a traveling high-field domain in the channel^3, 4, 30^ Finally, we note the *significant hysteresis in the drain current*, the value of which is significantly suppressed when returning from large drain voltage, compared to sweeping up. The nature of this hysteresis is very different to that exhibited by the fully-annealed devices (see Fig. 2(d)), being most pronounced over the very range of drain voltage for which the region of negative differential conductance emerges, and almost completely absent at larger *V*_*d*_. This should be contrasted with the hysteresis seen in Fig. 2(d), which is less prominent in nature, persists over a wider voltage range, and shows no systematic variation with drain voltage. The hysteresis in Fig. 3(a) is instead consistent with transferred-electron phenomenology; as the voltage is lowered back from its maximal extent, a significant number of carriers may remain trapped in the lower-mobility valley (see the discussion that follows below), so that it is only by fully reducing the voltage to zero that the carriers may be returned to their original valley. Collectively, these three distinct observations provide strong evidence for a Gunn effect, arising from hot-electron transfer between the T- and K-valleys^3, 4^

While the evidence of negative differential conductance in Fig. 3(a) is convincing, an apparent contradiction arises from the fact that no such behavior is present whatsoever in Fig. 2(d). The critical difference between these results comes from the fact that the various curves of Fig. 2(d) were obtained after fully annealing the device, until no further significant increase in its current level could be achieved (a process that typically required as much as 12 hours, as suggested by the data of Fig. 2(c)). In this fully annealed state, transistor currents in the range of µA/µm could reliably be achieved for appropriate gate and drain biasing. The data exhibiting negative differential conductance (Fig. 3(a)), on the other hand, were obtained by subjecting the device to only modest annealing (anneal times of around an hour or less), resulting in smaller drain currents (${\sim }$10 nA) for the same biasing conditions; this point may be appreciated by noting the lower current levels reached in Fig. 3(a) than in Fig. 2(d). In the discussion that follows, we propose a reconciliation of these very different behaviors, which considers the influence of mechanical strain on the band structure of WS_2_, and the manner in which this affects the negative differential conductance.

In the normal (unstrained) crystalline state of monolayer WS_2_, the energy separation between its T and K valleys is thought to be around 80 meV. With this relatively small difference, carriers are transferred to the T valleys at vanishingly-small fields and we consequently obtain no negative differential conductance. This point is demonstrated in the inset to Fig. 3(c), which shows the velocity-field characteristic calculated for unstrained monolayer WS_2_. (The calculations are based on an ensemble Monte Carlo approach, in which scattering between the K and T valleys is generated by T-point LO phonons, see Supplementary Information for further details.) Data are plotted for two different densities, neither of which exhibit any evidence of negative differential velocity. In contrast, it has recently been shown that biaxial strain raises the energy of the T valleys relative to that the K valleys^31, 32^ The resulting energy shift is relatively linear in the strain, and reduces the number of carriers in the T valley at thermal equilibrium. This in turn provides favorable conditions for the observation of negative differential conductance, by suppressing the likelihood of carriers being transferred into the T valleys at low fields^4^ In our simulations, we have systematically raised the T valleys and find that negative differential conductance begins when the inter-valley separation (Δ) is as little as 100 meV (i.e. when it is increased by just 25% relative to the unstrained case), corresponding to a strain level of just 1%^31, 32^ This estimate is consistent with recent scanning-microscopy studies of MoS_2_ FETs, which have revealed the presence of unintended strain in their channels, introduced during nanofabrication^33^ By assuming Δ = 110 meV, in Fig. 3(b) we compute the fraction of the carriers that remain in the K valley as a function of the electric field. Near zero bias, virtually all of the carriers are in the K valley, with a much smaller fraction accessing the T valley (as indicated schematically in Fig. 1(b)). With increasing electric field, however, the population of the K valley decreases rapidly (Fig. 3(b)), as carriers are driven into the T valley (as in Fig. 1(c)). This results in the emergence of pronounced negative differential velocity, as shown in the main panel of Fig. 3(c). Here, we overlay the velocity-field characteristics, calculated for two different carrier densities and for Δ = 110 meV, on top of one of the experimentally-measured transistor curves from Fig. 3(a). The data for both densities overlap well with the high-field portion of the experimental curve, consistent with the fact that appearance of negative differential conductance in Fig. 3(a) appears to be independent of the gate voltage. (Note that, since we do not know the carrier concentrations in the FET channel *per se*, it is not possible for us to infer a drift velocity from our transistor curves. Nonetheless, we note that the general shape of these curves is consistent with the expected variation of the drift velocity in Fig. 3(c) The negative differential conductance in the computed curves sets in beyond 20 kV/cm, again consistent with the experiment. While the peak-to-valley ratio of the negative differential conductance in Fig. 3(a) is considerably smaller than that found in GaAs^4^, it nonetheless appears sufficient to lead to instability and to domain propagation, as indicated by the relatively large hysteresis, and by the accompanying current noise, in the transistor curves.

### Avalanching Breakdown Under High Current Biasing

**Figure 4:**
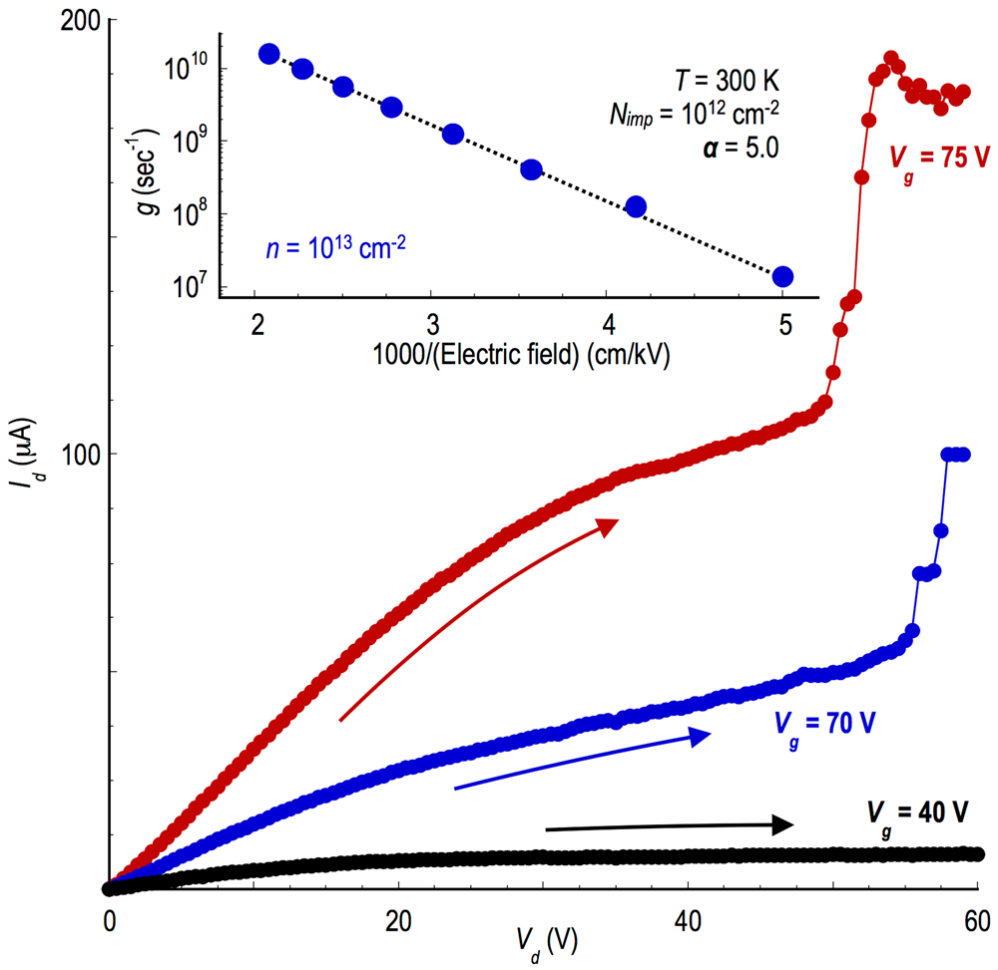
Avalanching breakdown in the WS2 FETs under strong electrical biasing. The main panel shows examples of current avalanching under high-current conditions. In these measurements, performed on Device A, a combination of thermal annealing and strong gate biasing is used to attain a large drain current ($\sim$10 µA), allowing the onset of avalanching. The inset to the figure plots the carrier generation rate (*g*) as a function of inverse electric field, calculated for Device A using the approach described in the Supplementary Information. The dotted line is a guide to the eye.

Negative differential conductance is just one example of a hot-carrier phenomenon that may be exhibited by semiconductor devices. Another well-known such effect is current breakdown due to avalanching, which can arise in the presence of large electric fields, once the drive current reaches an appropriate level. As noted already, the negative differential conductance discussed here was observed while the channel current in the FETs remained relatively low (<1 µA). By fully annealing the devices to optimize their current-carrying capacity, and utilizing both large drain and gate voltages, however, we are able to observe strong evidence of current avalanching. This phenomenon can be seen in Fig. 4, and occurs once the two bias voltages are increased such that the current rises to around 50–100 µA. (The avalanching is thus restricted to well-annealed devices, since these are the only ones capable of supporting the required current levels.) Focusing on the transistor curves obtained for *V*_*g*_ = 70 & 75 V in this figure, these do not exhibit current saturation but rather cross over to a slower increase as the drain bias is increased beyond the linear regime. With further increase of the bias, a sudden increase in the current is eventually observed, behavior that is indicative of avalanching. The capacity of the avalanching to destroy the device has meant that we have only been able to study its essential aspects, but it is clear already from Fig. 4 that it onsets at smaller drain bias as the gate voltage (and so the carrier density in the channel) is increased. A similar breakdown to that shown in Fig. 4 was also observed in other devices, for consistent values of the drain and gate voltages, once annealing had been used to maximize the current-carrying capacity of these transistors (see Supporting Information for additional examples).

**Figure 5:**
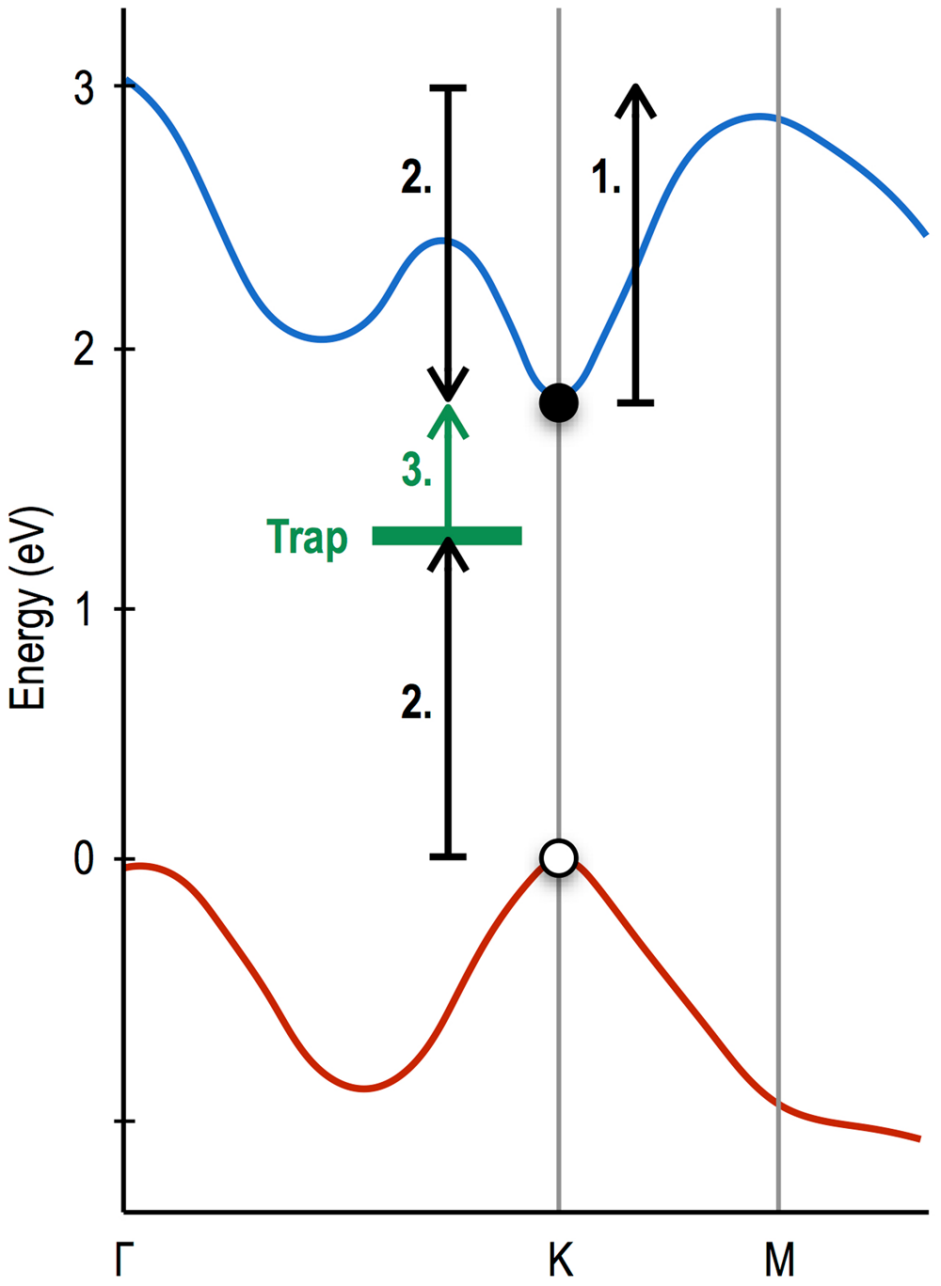
Schematic of the trap-assisted inverse Auger scattering process proposed for impact ionization. In Step 1, a carrier is driven up the conduction band by the strong electric field in the channel of the FET. In Step 2, the excited electron scatters from another electron in the valence band, losing its excess energy. This energy is transferred to the valence-band electron, which is excited into a mid-gap trap. Next, in Step 3, a second hot electron in the conduction band collides with the trapped electron, exciting it from the trap into the conduction band. At the end of this two-step process, the net result is the creation of an additional electron in the conduction band and a hole in the valence band (both indicated).

We have studied the details of carrier generation in WS_2_ using our ensemble Monte Carlo simulations, and find that the origin of the generation is very different to that in more-conventional semiconductors. The latter are characterized by relatively wide bands, which allow hot carriers to reach an energy several times that of the band gap. The impact ionization responsible for avalanching then occurs via a so-called “inverse Auger” process, in which a single hot electron interacts with a valence-band electron via the Coulomb interaction. (Another possibility, of course, is inter-band tunneling, but this is considered unlikely here since it generally produces a softer breakdown than the sharp current rise seen in our experiments. This is not altogether surprising, if we consider the long channel length of our devices, which is typically greater than 1 µm, and the large bandgap of monolayer WS_2_; these factors should combine to suppress the role of inter-band tunneling.) Providing that the conduction-band electron has sufficient energy, it is then able to excite the valence electron into the conduction band, creating an additional electron and a hole^34^ In the transition-metal dichalcogenides, however, the conduction band (as well as the valence band) is typically quite narrow, comparable to the size of the band gap itself^5^ Consequently, hot electrons are unable to gain sufficient energy for the inverse Auger process. It is known, however, that transition-metal dichalcogenides typically have a large number of mid-gap traps, which arise from defects (such as chalcogenide vacancies^35, 36^) that can mediate electron-hole recombination. One possible mechanism that we hypothesize as the source of the breakdown is the inverse of this process, in which an energetic electron initially excites a valence electron into a mid-gap trap, following which it is excited into the conduction band through its interaction with a second hot electron. While the exact distribution of the mid-gap states involved in this second-order process is not known for these devices, the carrier ionization should ultimately be determined by an integration over all intermediate energies. For this reason we feel that the breakdown should not depend crucially upon the density of traps, although it would, of course, disappear if there were no such traps. The essential point is that this two-step process, which we refer to as trap-assisted inverse Auger scattering, has the same effect of creating a new electron-hole pair (see Fig. 5). Since two electrons are required to initiate the process, we assume that the ionization is second-order and construct a scattering process similar to that in normal 2D materials (see the Supplementary Information for further details). By associating the experimentally observed breakdown in Fig. 4 with the avalanching threshold, we are able to calculate the electron-hole pair generation rate (*g*) and its dependence on electric field. This dependence is plotted in the inset to Fig. 4, where we have assumed an impurity concentration at the SiO_2_ interface of *N*_*imp*_ = 10^12^ cm^−2^, and an equilibrium electron density of 10^13^ cm^−2^. According to these calculations, the trap-assisted breakdown gives rise to a behavior in which the log of the generation rate varies linearly with the inverse electric field. This behavior corresponds to the “lucky” electron model suggested by Shockley^37, 38^, and gives rise to the soft breakdown behavior that is apparent in the main panel of Fig. 4. According to the calculations in the inset, the exponential growth in the generation rate occurs in the field range of 200–500 kV/cm. This should be compared with the results of our experiment, which show that the breakdown occurs at a drain bias close to 50 V (when *V*_*g*_ = 75 V), corresponding to an average field in the channel of around 230 kV/cm. Since the device is operated in saturation when the breakdown occurs, however, the peak field near the drain will likely be much higher than this, further promoting the trap-assisted avalanching.

## Discussion

In this Article, we have demonstrated important manifestations of hot-carrier transport in monolayer WS_2_ FETs. Negative differential conductance was observed, and was found to exhibit characteristics typical of the Gunn effect in more-traditional semiconductors. Reminiscent of those materials, the negative differential conductance was accompanied by the observation of pronounced hysteresis in the transistor characteristics, behavior that is consistent with the excitation of hot carriers from the lighter-mass K valleys into the heavier-mass T valleys. Simultaneous with the emergence of the negative differential conductance, the noise level in the drain current was also found to increase, a feature that suggests the formation of travelling, high-field domains within the FET channel. Overall, observation of the negative differential conductance was found to be sensitive to thermal annealing, a result that we have explained within a scenario in which the annealing relaxes unintentional strain within the transition-metal dichalcogenide channel. By reducing the K-T valley separation (Δ), this suppresses the negative differential conductance, as larger numbers of carriers populate the T valleys at thermal equilibrium, and thereby mask the influence of any transferred carriers at high electric fields. By fully annealing the transistors, and by employing strong gate biasing, we were ultimately able to achieve drive currents in excess of 10 μA/μm, under which conditions an avalanching breakdown of the drain current was observed. Since the narrow (conduction- and valence-) band width in this material is unable to support avalanching via the usual inverse Auger process, we have proposed instead a mechanism based on a two-stage, trap-assisted inverse Auger scattering. The results presented here suggest the strong potential of WS_2_ for use in various active devices. While further work still needs to be carried out, the instabilities associated with the negative differential conductance in this material may find application in compact microwave or terahertz sources, while the high-field breakdown may be useful in different photonic sensors.

## Methods

WS_2_ crystals were grown by CVD on commercial Si/SiO_2_ wafers (as described in refs. 8 and 9), featuring pre-defined Cr/Au (5-nm/45-nm) markers that allowed alignment to individual WS_2_ crystals to be performed in subsequent electron-beam lithography steps. Ambient Raman spectra of the crystals were obtained using a Renishaw inVia Raman Microscope, using a laser excitation of wavelength 488-nm and providing access to Raman shift in the range of 100–4000 cm^−1^. Electrical measurements of the WS_2_ FETs were performed after first wire bonding them into a ceramic DIP package, which was then mounted into the enclosed vacuum space of a variable-temperature system, capable of heating the devices to 500 K. This system allowed thermal annealing of the samples to be performed under the temperature conditions described in the main text, during which time the sample space was maintained at a vacuum of approximately 10^−5^ mbar. Transistor curves of the devices were recorded using a Keithley 2400 source-measure unit, with a second, similar, instrument being used to source the gate voltage. In measurements of the avalanching breakdown in these devices, a programmed compliance level was used to ensure that the drain current did not exceed 1 mA, thereby limiting irreversible damage to them.

## Electronic supplementary material

Supplementary Information
