# Supplementary material for: Negative Differential Conductance & Hot-Carrier Avalanching in Monolayer WS2 FETs
Source: Sci Rep. 2017 Sep 12;7:11256. doi: 10.1038/s41598-017-11647-6 (PMC5595880; doi:10.1038/s41598-017-11647-6)
Supplement: Supplementary file 1 — Supplementary Information [file 41598_2017_11647_MOESM1_ESM.pdf]

Supplementary Information

Negative Differential Conductance & Hot-Carrier Avalanching in Monolayer WS_2 FETs

G. He, J. Nathawat, C.-P. Kwan, H. Ramamoorthy, R. Somphonsane, M. Zhao, K. Ghosh,

U. Singisetti, N. Perea-López, C. Zhou, A.L. Elías, M. Terrones, Y. Gong, X. Zhang,

R. Vajtai, P. M. Ajayan, D. K. Ferry & J. P. Bird

1. Device Details

WS_2 crystals were grown on commercial silicon wafers by chemical vapor deposition (CVD). This yielded triangular-shaped single crystals, a few microns on a side, whose monolayer character was confirmed by Raman spectroscopy (see Fig. 2(b) of the main paper). Source and drain contacts were formed by depositing 5 nm of Cr, followed by 50 nm of Au. Gating of the transistors was achieved by biasing the heavily-doped Si substrate, separated from the WS_2 crystals by a 280-nm thick SiO_2 dielectric layer. Some twenty different transistors were fabricated for the purpose of this study, with channel lengths varying from 1 – 2 µm. Table S1 below provides the dimensions of the different devices (A – C) whose results are presented in the main paper.

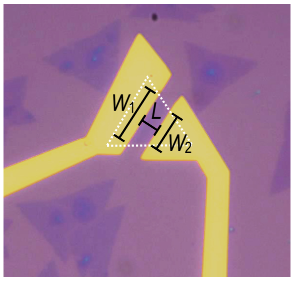

Device	W_1 (µm)	W_2 (µm)	L (µm)
A	5.7	3.6	2.0
B	3.7	3.7	1.0
C	4.7	4.8	1.8

Table S1. Identification of the different devices used in this study. Labeling scheme used to identify, and the dimensions of, the different devices investigated in the main paper. Shown left is the device (Device A) of Fig. 2(a) of the main paper, with its key dimensions indicated. The table on the right indicates these dimensions for Devices A – C.

While the main focus of the main paper is on the high-field properties of the transistors, we have also studied their low-field mobility, by utilizing the features of their transconductance. While not too much faith may be placed in these measurements, due to the influence of contact resistance,[S1] inferred values at room temperature are typically in the range of 1 – 10 cm^2/Vs, consistent with other studies.[S2]

2. Supporting Experimental Results

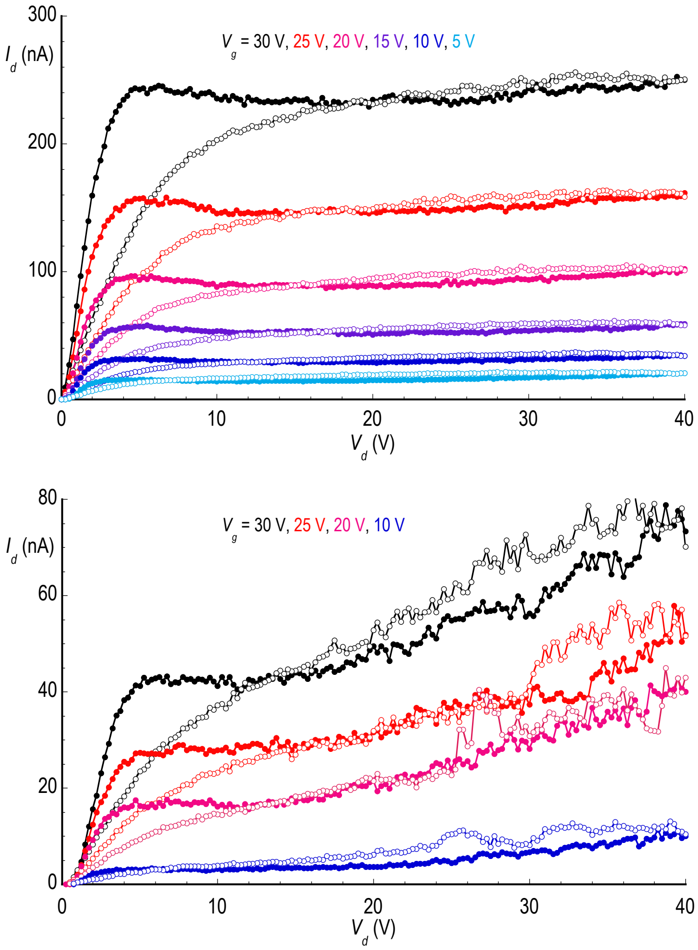

Fig. S1. Other examples of negative differential conductance and associated instabilities. Both plots were obtained under the same measurement conditions as described in the main paper, following modest annealing for around an hour. The upper plot was obtained for Device A, the bottom one for Device B. Filled symbols correspond to up sweeps of the drain voltage, open ones to down sweeps.

In Fig. S1 we show examples of negative differential conductance measured in other devices. As with the results presented in the main manuscript, these data were obtained for partial annealing of the device (anneal times around 1 hour), for which the drain-current level remains in the sub-μA range. The data shown here exhibit clear similarities with those presented in the manuscript. Negative differential conductance is observed, along with pronounced hysteresis as a function of sweep direction and enhanced noise. In the lower panel, the overall current level remains below 100 nA and the linear slope to the current at large drain voltages has the effect of "pulling up" the region of negative differential conductance so that it is less pronounced. Nonetheless, in spite of these device-dependent variations, the similarity in behavior between Figs. 3(a) and Fig. S1 is, we believe, very clear.

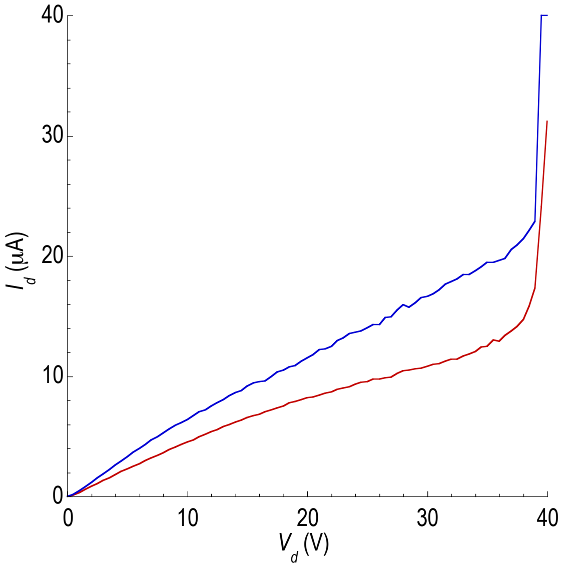

Fig. S2. Other examples of breakdown in the devices. The blue line was obtained for a device with W_1 = 3.5 μm, W_2 = 1.8 μm and L = 1.6 μm, at a gate voltage of 20 V. The red line was obtained for a device with W_1 = 4.0 μm, W_2 = 2.1 μm and L = 2.2 μm, at a gate voltage of 25 V.

In Fig. S2 we show some further examples of breakdown behavior in two different WS_2 FETs. These transistors are similar in structure to Devices A – C but were destroyed by the avalanching process shown in the figure. In spite of this, we note many similarities with the behavior reported in Fig. 4 of the

main manuscript; the breakdown occurs once the channel current is in the range of 20 μA, for a drain voltage of around 40 V and with the gate voltage in the range of 20 – 25 V.

3. Modeling Details

WS_2 is a layered compound with an indirect band gap in bulk, which becomes direct in the monolayer limit. The band extrema for the monolayer are located at the K points of the Brillouin zone, so that there are two minima (K and K′) for the conduction band. Subsidiary valleys of the conduction band arise from the residual valleys of what was the indirect gap and are referred to as the T valleys. The latter lie midway between the Γ and K point, on the line that connects them. The conduction and valence bands are derived from the transition-metal *d* states, and are consequently very narrow and nonparabolic. The nonparabolicity leads to a mass enhancement factor $m_{eff} = m^*(1 + 2\alpha E)$, where α = 5 eV^{-1} is the mass enhancement factor, and m^* is the band edge effective mass. The value of this mass is approximately $0.32m_o$ in the K valleys and $0.75m_o$ in the subsidiary T valley (which lies only approximately 80 meV above the K minimum in the unstrained state). These values are relevant near the bottom of their respective valleys, and so, in the presence of the aforementioned nonparabolicity, are not directly related to any effective mass that might be inferred from the computed velocity saturation. As for the phonon spectra in WS_2, these are quite similar to those of MoS_2, although the coupling constants are not nearly as well known. Consequently, we have adapted the values from MoS_2[S3] by scaling from the known lattice spacings and atomic masses.

To study the details of high-field transport in WS_2, we have solved the Boltzmann transport equation via an ensemble Monte Carlo method.[S4,S5] In this treatment, scattering of carriers in the K valleys, and between them, is dominated by the acoustic phonons and the homopolar and LO optical phonons, which are scattered via the deformation potential interaction. These are the Γ (intravalley) and K (equivalent intervalley via the LO) phonons. Scattering between the K valleys and the T valleys is via T point LO phonons. Similarly, the scattering between the various T valleys is by these same phonons. The coupling constants for the various phonon modes have been adapted from DFT calculations of MoS_2 by

Kaasbjerg *et al.*[S3] A potential issue here arises from the influence of the polar LO modes, which one would expect to be present in the monolayer. Scattering by these modes has the same basic energy dependence as the nonpolar ones, so can be included by a modification of the coupling constant for the latter process. A potential weakness of this approach is that the role of nonequilibrium phonons (both optical and acoustic) is not addressed in the simulations. Nonetheless, in spite of this omission, our simulations confirm the presence of negative differential conductance due to intervalley transfer. The obtained agreement with experiment suggests that the influence of nonequilibrium phonons should largely be a higher order effect. Nonetheless, a fully complete microscopic model should properly account for the role of nonequilibrium phonons, including the decay of nonequilibrium optical modes into the acoustic ones that ultimately transfer energy to the heat sink.

In addition to the influence of the various phonon modes, our Monte-Carlo calculations also account for Coulomb scattering from remote ionized impurities in the SiO_2 gate dielectric, and from the polar surface modes of the SiO_2. These impurities are taken to be present at a nominal density in the range of $0.3 – 1.0 \times 10^{12}$ cm^{-2}, with a uniform distribution on the surface of the oxide.

A novel concept introduced in the main paper is of the idea of a trap-assisted inverse Auger process that may serve as a pathway to avalanching at high electric fields. Whereas the normal inverse-Auger interaction varies as $(E – E_T)$, where $E_T > E_g$ is some threshold energy, we expect the trap-assisted (second-order) process to vary as $(E – E_T)^2$. However, as we have no solid theory for the two step interaction, we have to take experiment to suggest its strength. Breakdown is observed to occur at a given electric field, which means that the ionization coefficient at that field should satisfy $\alpha(\mathcal{E}_{BD})L \geq 1$, where $\mathcal{E}_{BD}$ is the breakdown electric field and L is the FET channel length. The impact ionization process is characterized by a generation rate (per carrier per second) for ionizing collisions, which is related to the ionization coefficient as $g(\mathcal{E}) \sim \alpha(\mathcal{E})v_d(\mathcal{E})$, with v_d the electron drift velocity. Thus, having an observed breakdown field from experiment, we can adjust the ionizing collision rate to yield an appropriate value for the generation rate at that field. We may then examine the generation rate at other fields without

other approximations. This is what is plotted in the inset to Fig. 4 of the main paper, based on an analysis of the experimental data in the main panel of that figure.

References

S1. Hwang, W. S., Remskar, M., Yan, R., Protasenko, V., Tahy, K., Chae, S. D., Zhao, P., Konar, A., Xing, H., Seabaugh, A., Jena, D. Transistors with chemically synthesized layered semiconductor WS_2 exhibiting 10^5 room temperature modulation and ambipolar behavior. *Appl. Phys. Lett.* **101**, 013107 (2012).

S2. Wang, Q. H., Kalantar-Zadeh, K., Kis, A., Coleman, J. N., Strano, M. S. Electronics and optoelectronics of two-dimensional transition metal dichalcogenides. *Nature Nanotechnol*. **7**, 699 – 712 (2012).

S3. Kaasbjerg. K., Thygesen, K. S., Jacobsen, K. W. Phonon-limited mobility in *n*-type single-layer MoS_2 from first principles. *Phys. Rev*. B **85**, 115317 (2012).

S4. Ferry, D. K. *Semiconductors* (Macmillan, New York, 1991), pp. 354 – 370.

S5. Ferry, D. K. Electron transport in some transition metal di-chalcogenides. *submitted for publication.*
